# Expansion of Knowledge on OCT1 Variant Activity *In Vitro* and *In Vivo* Using Oct1/2^−/−^ Mice

**DOI:** 10.3389/fphar.2021.631793

**Published:** 2021-02-15

**Authors:** Bridget L. Morse, Lisa Hong Chen, John T. Catlow, John K. Fallon, Philip C. Smith, Kathleen M. Hillgren

**Affiliations:** ^1^ Drug Disposition, Eli Lilly and Company, Indianapolis, IN, United States; ^2^ Division of Pharmacoengineering and Molecular Pharmaceutics, Eshelman School of Pharmacy, University of North Carolina at Chapel Hill, Chapel Hill, NC, United States

**Keywords:** drug transport, organic cation transporter 1 (OCT1), pharmacokinetics, knockout mice, metabolite kinetics, targeted proteomics, pharmacogenetics

## Abstract

The role of organic cation transporter 1 (OCT1) in humans is gaining attention as data emerges regarding its role in physiology, drug exposure, and drug response. OCT1 variants with decreased *in vitro* function correlate well with altered exposure of multiple OCT1 substrates in variant carriers. In the current research, we investigate mechanisms behind activity of OCT1 variants *in vitro* by generating cell lines expressing known OCT1 variants and quantifying membrane OCT1 protein expression with corresponding OCT1 activity and kinetics. Oct knockout mice have provided additional insight into the role of Oct1 in the liver and have reproduced effects of altered OCT1 activity observed in the clinic. To assess the complex effect of Oct1 depletion on pharmacokinetics of prodrug proguanil and its active moiety cycloguanil, both of which are OCT1 substrates, Oct1/2^−/−^ mice were used. Decreased membrane expression of OCT1 was demonstrated for all variant cell lines, although activity was substrate-dependent, as reported previously. Lack of change in activity for OCT1*2 resulted in increased intrinsic activity per pmol of OCT1 protein, particularly for sumatriptan but also for proguanil and cycloguanil. Similar to that reported in humans with decreased OCT1 function, systemic exposure of proguanil was minimally affected in Oct1/2^−/−^ mice. However, proguanil liver partitioning and exposure decreased. Cycloguanil exposure decreased following proguanil administration in Oct1/2^−/−^ mice, as did the systemic metabolite:parent ratio. When administered directly, systemic exposure of cycloguanil decreased slightly; however liver partitioning and exposure were decreased in Oct1/2^−/−^ mice. Unexpectedly, following proguanil administration, the metabolite ratio in the liver changed only minimally, and liver partitioning of cycloguanil was affected in Oct1/2^−/−^ mice to a lesser extent following proguanil administration than direct administration of cycloguanil. In conclusion, these *in vitro* and *in vivo* data offer additional complexity in understanding mechanisms of OCT1 variant activity as well as the effects of these variants *in vivo*. From cell lines, it is apparent that intrinsic activity is not directly related to OCT1 membrane expression. Additionally, in situations with a more complicated role of OCT1 in drug pharmacokinetics there is difficulty translating *in vivo* impact simply from intrinsic activity from cellular data.

## Introduction

In humans, organic cation transporter 1 (OCT1) is localized in the liver and intestine, organs pertaining to drug absorption, distribution, metabolism and excretion (Drozdzik et al., 2019). As many therapeutic agents are demonstrated OCT1 substrates (Koepsell, 2020), the potential exists for a role of OCT1-mediated transport on the disposition of therapeutic drugs. This prospect was propagated by the identification of OCT1 variants with varying degrees of transport activity impacting cellular exposure, initially on metformin uptake but followed by sumatriptan, fenoterol, proguanil, ranitidine and others (Shu et al., 2007; Matthaei et al., 2015; Meyer et al., 2017; Tzvetkov et al., 2018; Matthaei et al., 2019; Jensen et al., 2020). Subsequent clinical evaluation in subjects carrying these variant alleles has demonstrated clinically relevant effects on the exposure of these therapeutic agents (Matthaei et al., 2015; Tzvetkov et al., 2018; Matthaei et al., 2019). Interestingly, OCT1 pharmacogenetics demonstrate substrate-specificity, most notably OCT1*2, for which uptake of some substrates, such as fenoterol and metformin, is impaired compared to wildtype, while for sumatriptan, proguanil and cycloguanil activity was relatively maintained (Shu et al., 2007; Matthaei et al., 2015; Matthaei et al., 2019).

Due to the observed clinical relevance of OCT1 variants on the pharmacokinetics of OCT1 substrates, tools for identifying the role of OCT1 in the pharmacokinetics of an investigational drug have become important. Notably, *in vitro* data on the uptake activity of various OCT1 variants has correlated quite well with *in vivo* observations. A prominent example is the effect of OCT1 activity on the metabolite ratio for cycloguanil:proguanil, in which the authors were able to demonstrate a continuous correlation of *in vitro* activity to the relationship observed *in vivo* (Matthaei et al., 2019). Oct knockout mice have also provided insight into the role of Oct1 in hepatic clearance and partitioning, as well as its physiologic role in lipid metabolism (Higgins et al., 2012; Liang et al., 2018; Morse et al., 2020). We previously assessed Oct1/2^−/−^ mice as a model for reproducing or predicting the effect of OCT1 variants in the clinic. In these studies, hepatic clearance of sumatriptan and fenoterol was significantly decreased, and the change in oral and IV clearance was similar to that reported in human carriers of OCT1 null variants (Morse et al., 2020). In these mice, a corresponding decrease in liver partitioning was determined for sumatriptan and fenoterol and was also previously demonstrated for metformin (Higgins et al., 2012). We did not find this Oct1/2^−/−^ mice model to be as robust for the effects of Oct1 deficiency on ondansetron or tropisetron pharmacokinetics, although the clinical data for comparison in humans is also not as robust as that for other OCT1 substrates mentioned above (Tzvetkov et al., 2012).

In the current research, we generated cell lines expressing 8 OCT1 variants proteins using a novel stable lentiviral transfection method and confirm previous results for activity toward OCT1 substrates sumatriptan, fenoterol, metformin, proguanil and cycloguanil. We additionally quantitated the OCT1 membrane protein expression level of each one of these variants, which has not previously been reported, to understand changes in substrate kinetics relative to protein OCT1 expression. To follow-up on previous application of the Oct1/2^−/−^ mouse model for sumatriptan, fenoterol and metformin, we assess the pharmacokinetics of proguanil and cycloguanil. A considerable advantage to the use of rodent models is the ability to assess tissue concentrations and to assess the effect of altered transport activity of pharmacokinetics of a metabolite administered directly. Therefore, we utilize the Oct1/2^−/−^ mouse model to assess the plasma pharmacokinetics of proguanil for comparison to that in humans, as well as cycloguanil following proguanil administration and administered directly. Additionally, we use the model to assess the liver exposure changes in these agents, as this is a site of action and may add to hypotheses on liver exposure of these therapeutic agents in patients with decreased OCT1 activity.

## Materials and Methods

### Generation of OCT1 Variant Cell Lines and Uptake of OCT1 Substrates

Generation of OCT1 variant cell lines was performed as reported previously for wildtype OCT1*1 (Morse et al., 2020). OCT1*2 (Met420del), OCT1*3 (Arg61Cys), OCT1*4 (Gly401Ser), OCT1*5 (Met420del and Gly465Arg), OCT1*6 (Cys88Arg and Met420del), OCT1*8 (Arg488Met) and OCT1*10 (Ser189Leu) were synthesized and cloned into the pLenti6.3 vector. pLenti6.3 empty vector and pLenti6.3- OCT1 variants were transfected into a lentiviral package cell line Lenti-X-293T to produce lentivirus supernatants. HEK293 cells were then transfected with these nine lentivirus supernatants respectively and OCT1 variants was selected by blasticidin (5 μg/ml) to generate stable cell lines. OCT1 expression in HEK293-OCT1*1 was confirmed by flow cytometry using antibody staining (Novusbio Cat#NBP1-51684). HEK-293 stably transfected cells with empty vector, OCT1 variants were grown in 5% CO2 at 37 °C in DMEM supplemented with 10% FBS, 50 μg/ml gentamicin, and 5 μg/ml blasticidin. Cell lines were maintained in T-75 flasks, reaching approximately 80% confluence before being passaged twice weekly at 1:10 ratio (volume: volume).

HEK293-VC (vector control) and -OCT1 expressing cells were seeded onto 12-well poly-D lysine plates at concentrations ranging from 1.7 × 10^5^ to 2.7 × 10^5^ cells/mL. Three days post-seeding, the cells were washed twice with prewarmed pH 7.4 HBSS buffer and preincubated with assay buffer for 10 min at 37 °C. Following the preincubation, cells were treated with the desired substrate for one or 2 min at 37 °C. After one or 2 min at 37 °C, the cells were washed three times with ice-cold HBSS and extracted with 80% MeOH containing an internal standard mix for sample analysis via LC-MS/MS. A separate set of cells were used to determine protein concentration by bicinchoninic acid method. Uptake was assessed in triplicate in two separate experiments. Using the same experimental method, a range of substrate concentrations was used to assess the kinetics of sumatriptan and fenoterol in OCT1*1 and OCT1*2 expressing cells, using a time point of 1 min at each concentration.

For OCT1 protein quantitation, the membrane fraction from each sample was extracted using an adapted differential surfactant extraction method (Qasem et al., 2020). For each variant, 2 separate samples were analyzed in duplicate, from cell passages one week apart. Quantitation of transporter expression was performed by nanoLC-MS/MS using SIL (stable isotope labeled) peptide standards as previously described (Khatri et al., 2019; Morse et al., 2020). The reporting peptide for the (human) OCT1 concentrations is LSPSFADLFR (the UniProt accession # is O15245). The peptide ENTIYLK was used as confirmatory. Concentrations of Na^+^/K^+^-ATPase were also measured by nanoLC-MS/MS, for use as a membrane marker control.

### Pharmacokinetics in Oct1/2^−/−^ Mice

The pharmacokinetics of proguanil and cycloguanil were assessed in Oct1/2^−/−^ mice as described previously for other OCT1 substrates (Morse et al., 2020). Studies were carried out at Covance (Greenfield, IN) and were approved by the Institutional Animal Care and Use Committee. Male Oct1/2^−/−^ mice were taken from a breeding colony, maintained by Taconic. Age-matched FVB male mice were also purchased from Taconic. To evaluate the blood pharmacokinetics, groups of mice (n = 5, ages 8–14 weeks) were administered proguanil (2, 10, and 30 mg/kg) or cycloguanil (2 mg/kg) intravenously via the tail vein and serial blood samples collected as dried blood spots. To evaluate tissue partitioning, groups of mice (*n* = four to five, ages 8–14 weeks) were administered proguanil (2, 10 and 30 mg/kg) or cycloguanil (2 mg/kg) and blood, plasma, and 4 tissues (liver, kidney, spleen, duodenum) collected at 0.75, 1.5, 2, 4 or 8 h post-dose. Tissues and plasma were kept at <60 °C until analysis for concentrations of proguanil and cycloguanil by LC/MS-MS (details in Supplemental Material). Both cycloguanil and proguanil were quantified in animals administered proguanil.

### Data Analysis

Uptake of OCT1 substrates in cells expressing OCT1 variants were normalized first by total protein, then represented as fold uptake of OCT1*1. The data were further analyzed by normalizing uptake by total amount of membrane OCT1 protein in each variant, then again represented as fold uptake of OCT1*1. K_m_ and V_max_ values for sumatriptan and fenoterol were determined using the equation below:

$$\text{Uptake}=\;\frac{{\text{V}}_{\max}\cdot \left[S\right]}{{\text{K}}_{m}+\left[S\right]}+{\text{P}}_{d}\cdot \left[S\right],$$

where V_max_ and K_m_ represent the maximal rate of uptake and the concentration and half maximal rate of uptake, P_d_ represents passive diffusion and [S] represents substrate concentration.

*In vivo* blood parameters in mice were determined by noncompartmental analysis using Watson 7.2. Clearance and liver partitioning of proguanil was dose-proportional from 2 to 30 mg/kg, therefore these groups were combined and data dose-normalized to 10 mg/kg. Metabolite ratio (M:P) in the plasma was calculated as cycloguanil AUC/proguanil AUC. Renal clearance (CLR) was determined as Ae/AUC, where Ae represents the amount recovered in urine, and AUC represents the area under the blood concentration–time curve (the mean AUC of animals administered the same dose of compound IV). The CLR was then corrected for creatinine recovery as described previously (Morse et al., 2020). Mean hepatic clearance was calculated as total clearance-CLR (determined as one value for each compound, due to pooled nature of urine samples). Tissue partitioning coefficients (Kp) at single timepoints were calculated as tissue/plasma concentrations. AUC in the liver was determined by noncompartmental analysis using the sparse sampling function in Phoenix 64. Liver metabolite ratio (M:P) was calculated as cycloguanil AUC/proguanil AUC. Student’s t-tests were used to determine significant differences in pharmacokinetic parameters or tissue Kp values between wildtype and knockout mice using GraphPad 9.3.

## Results

**FIGURE 1:**
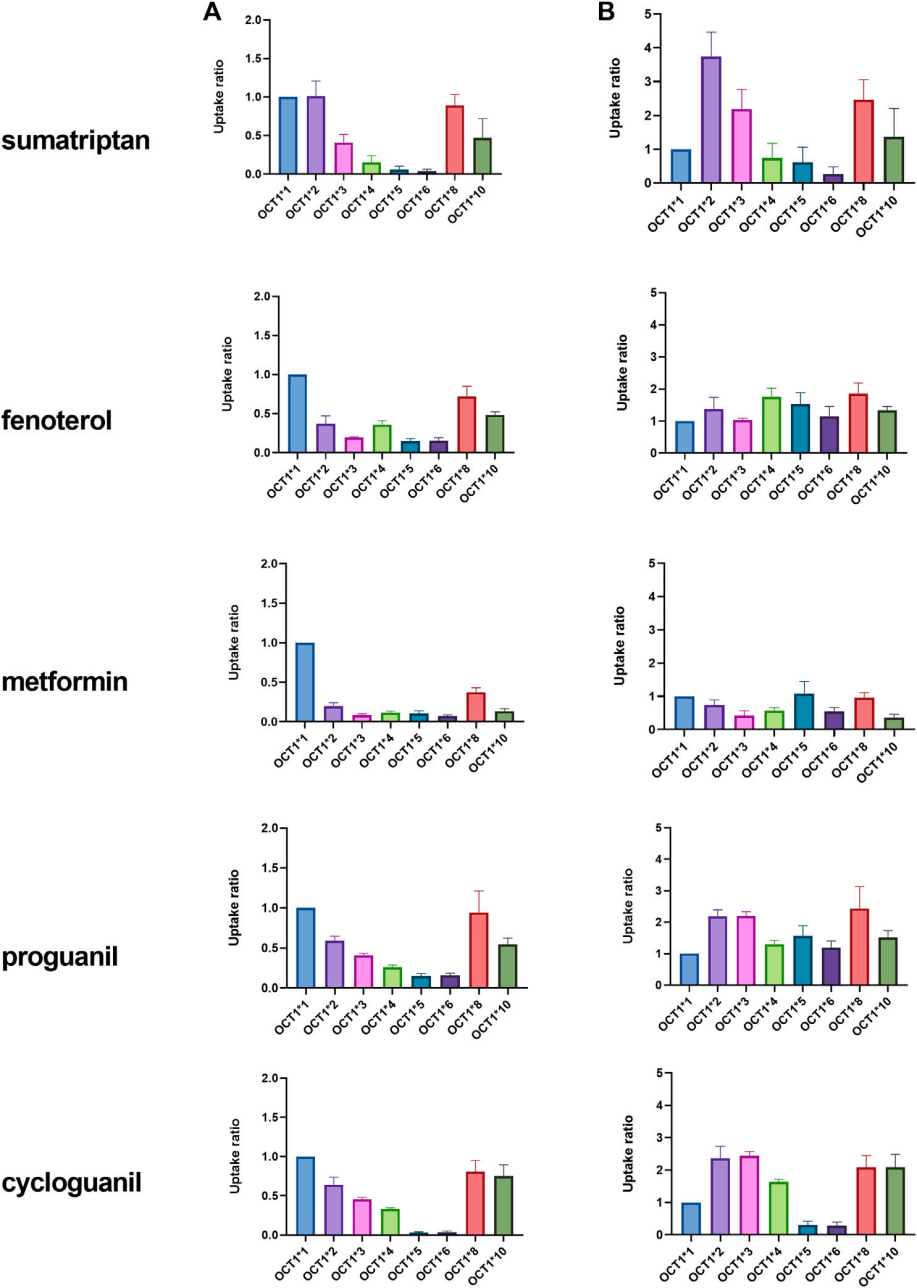
Uptake of OCT1 substrates in HEK cells expressing wildtype (*1) and variant OCT1 protein. Uptake was assessed over 1 or 2 min, depending on substrate, in triplicate, at substrate concentration of 3 μM, with the exception of metformin, which was assessed at 22 µM. Data are presented as fold uptake compared to OCT1*1 (mean ± SD). Data in column **(A)** represent uptake prior to normalization for absolute OCT1 membrane protein expression. Data in column **(B)** represent uptake after normalization for absolute OCT1 membrane protein expression measured in cells expressing each variant.

**FIGURE 2:**
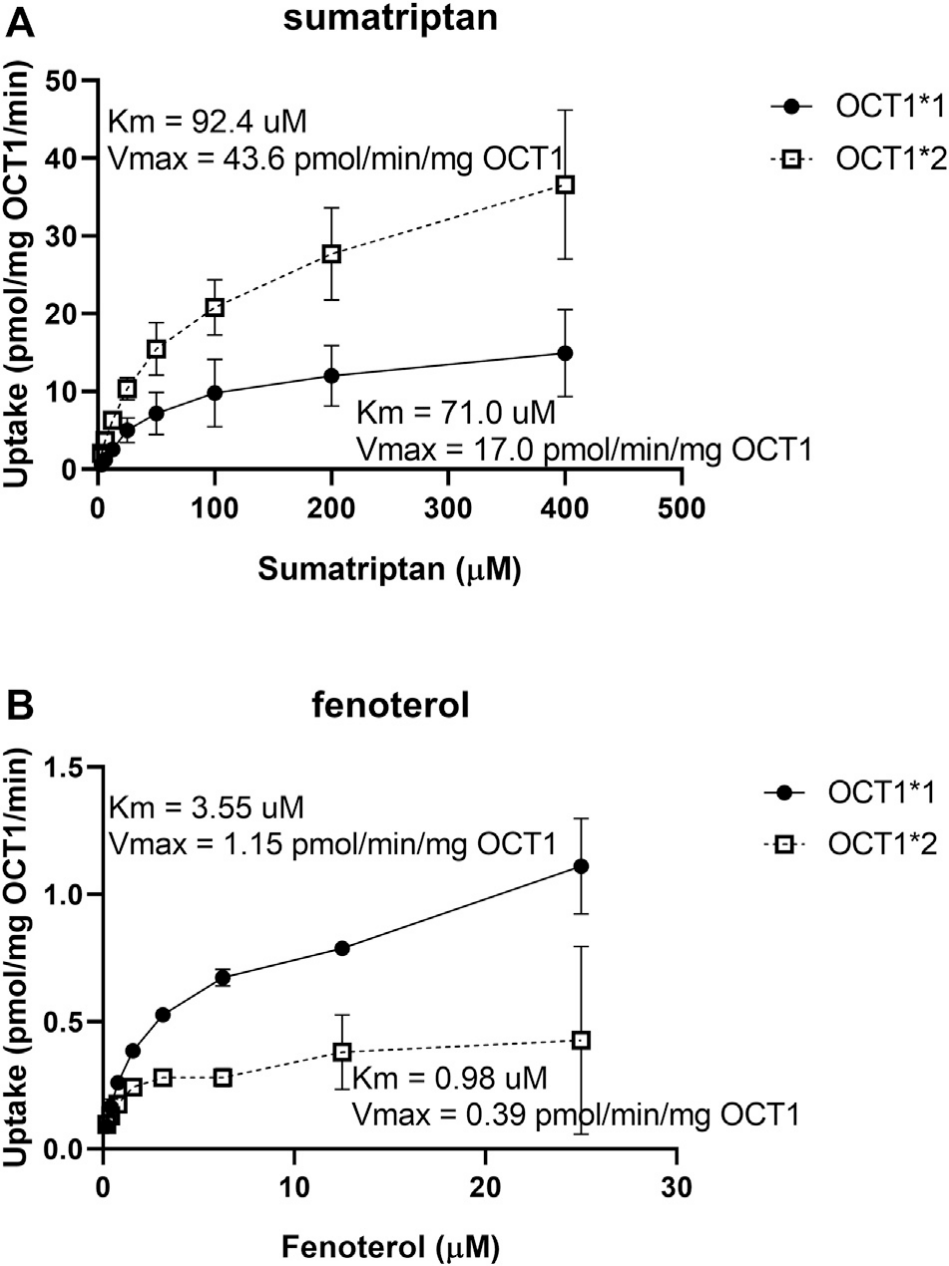
Kinetics of sumatriptan (A) and fenoterol (B) in HEK cells expressing OCT1*1 or OCT1*2. Uptake was assessed over 1 min, in triplicate, at substrate concentration of 3 µM. Data are presented as mean ± SD.

The uptake of known clinical OCT1 substrates in cell lines expressing wildtype and variant OCT1 protein are shown in Figure 1; uptake values are shown both before (A) and after (B) normalizing for measured membrane expressed OCT1 protein. In general, uptake for the substrates in the respective variants reproduce well those reported previously (Shu et al., 2007; Matthaei et al., 2015; Tzvetkov et al., 2018; Matthaei et al., 2019). The substrate-dependence of OCT1*2 previously demonstrated is clearly observed. Membrane OCT1 protein expression was lower in all variants tested compared to OCT1*1; absolute OCT1 concentrations from membranes of each variant cell line are shown in Supplementary Table S1, as well as the concentrations of membrane marker Na^+^/K^+^-ATPase in the cell samples, which were very similar for samples from each variant. Interestingly, after normalizing for measured OCT1 protein, increased intrinsic activity per pmol of OCT1 for certain substrates was evident, most notably sumatriptan for OCT1*2, whereas intrinsic activity for fenoterol and metformin in OCT1 variants appeared to be reconciled by normalization of membrane OCT1 expression. As two substrates with differing activity for OCT1*2, kinetics of sumatriptan and fenoterol in OCT1*1 and *2 were assessed. Prior to normalizing for membrane expressed OCT1 protein, for sumatriptan neither K_m_ nor V_max_ were dramatically different in OCT1*2 compared to OCT1*1 (mean K_m_ of 68.3 vs 98.9 µM and V_max_ of 5,360 vs. 4,300 pmol/min/mg, data not shown). However, as shown in Figure 2, after normalizing for OCT1 protein, the V_max_ of sumatriptan in OCT1*2 was 2.6-fold higher than that for OCT1*1 (43.6 vs. 17.0 pmol/min/mg,OCT1) while K_m_ values remained similar (93.4 vs 71.0 µM), consistent with higher intrinsic activity of OCT1*2 when normalized for OCT1 protein (Figure 1). For fenoterol, the V_max_ for OCT1*2 was decreased substantially compared to OCT1*1 prior to normalizing for membrane expressed OCT1 protein (351 vs 32.0 pmol/min/mg), while the K_m_ was affected to a lesser extent (3.24 vs. 0.85 µM). After normalizing for OCT1 protein, the K_m_ and V_max_ were similarly ∼3-fold lower in OCT1*2 compared to OCT1*1 (mean K_m_ of 3.55 vs 0.98 µM and V_max_ of 1.15 vs. 0.39 pmol/min/mg,OCT1), consistent with maintained intrinsic activity of OCT1*2 when normalized for OCT1 protein (Figure 1).

**FIGURE 3:**
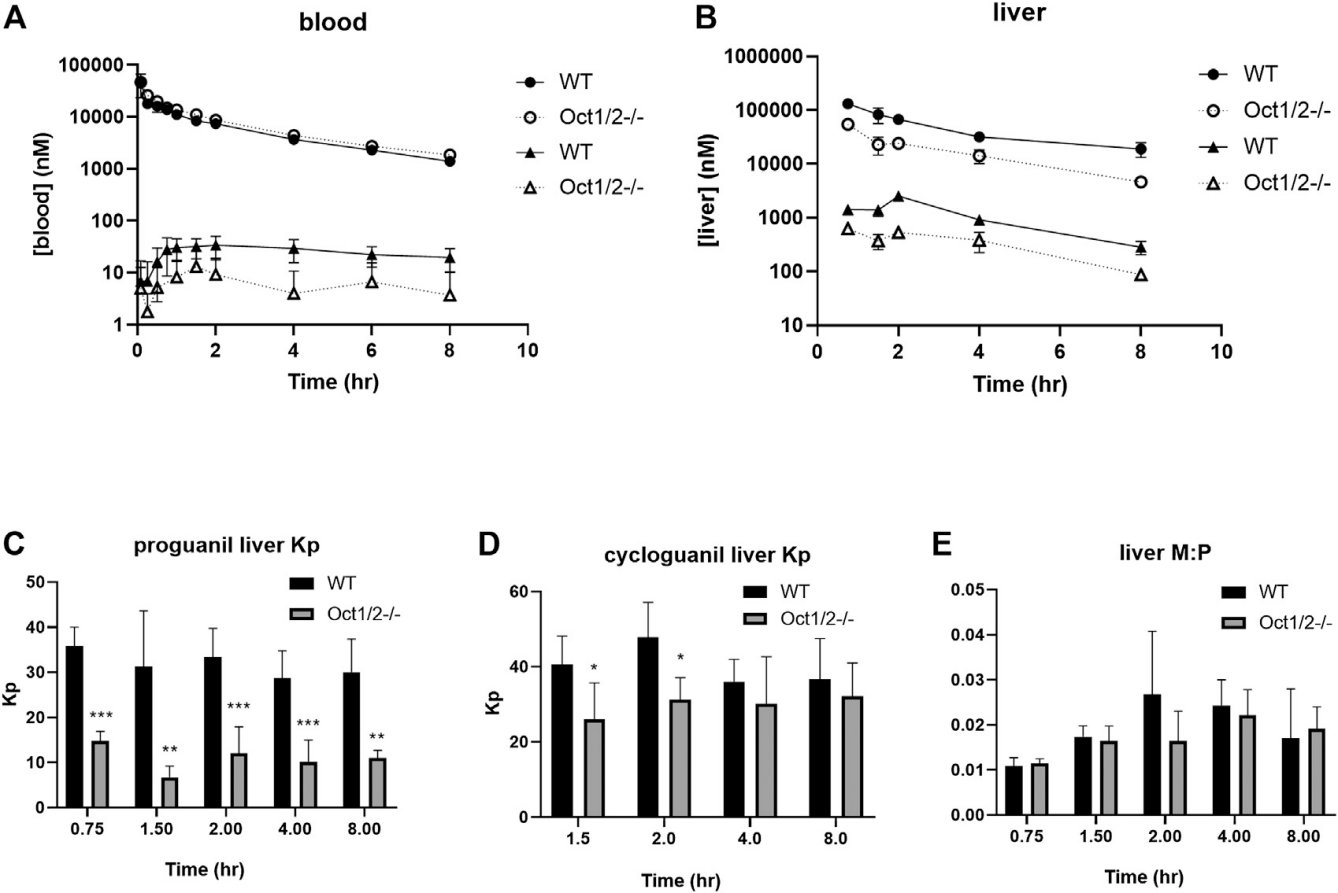
**Pharmacokinetics of proguanil/cycloguanil in wildtype (WT) and Oct1/2** ^**−/−**^ **mice following IV administration of proguanil.** Shown are dose-normalized blood **(A)** and liver **(B)** concentrations for mice (*n* = 5 or 5/timepoint) administered proguanil 2, 10 or 30 mg/kg. Filled symbols represent wildtype mice and open symbols represent knockout mice. Circles represent concentrations of proguanil and triangles represent concentrations of cycloguanil **(C** and **D, E)** Liver Kp and M:P values determined from tissues collected at 0.75 and 2 h (2 mg/kg), 1.5 and 4 h (10 mg/kg) or 8 h (30 mg/kg) post-dose. Data presented as mean ± SD. *p < 0.05 using student's t-test, compared to WT. **p < 0.01 using student’s t-test, compared to WT. ***p < 0.001 using student’s t-test, compared to WT. Kp = tissue:plasma partition coefficient. M:P = metabolite:parent ratio.

The pharmacokinetic parameters of OCT1 substrates proguanil and cycloguanil in WT and Oct1/2^−/−^ mice are given in Table 1. The blood concentration-time profiles are shown in Figure 3A. The clearance of proguanil was minimally, but significantly decreased in wildtype mice compared to Oct1/2^−/−^ mice. Accordingly, proguanil was found to be excreted primarily in the urine; renal clearance was similar between wildtype and knockout mice. Liver partitioning and exposure of proguanil was, however, substantially affected by Oct knockout, as shown in Figures 3B,C, and liver Kp and AUC decreased by ∼3-fold (Table 1).

**TABLE 1:** **Effect of Oct knockout on the pharmacokinetics of proguanil and cycloguanil following IV administration of both proguanil and cycloguanil.** Shown are dose-normalized blood and liver pharmacokinetic parameters for mice (*n* = 5 or 5/timepoint) administered proguanil 2, 10 or 30 mg/kg or cycloguanil 2 mg/kg. Data presented as mean ± SD. Ratio of KO/WT are reported below mean values with significant changes.

	Proguanil		Cycloguanil		Cycloguanil	
Compound dosed		Proguanil			Cycloguanil	
	WT	KO	WT	KO	WT	KO
AUC_blood_ (nM^*^Hr)	46,300 (6,420)	61600^ccc^ (678)	274 (33.5)	92.8^bb^ (12.4)	3,315 (715)	4,815 (1,570)
	1.33		0.34		1.43	
CL (ml/kg/min)	13.8 (2.24)	10.1^ccc^ (1.15)	—	—	41.2 (9.20)	30.1^a^ (8.96)
	0.73				0.73	
M:P	—	—	0.00504 (0.00041)	0.00147^ccc^ (0.000064)	—	—
			0.29			
CLrenal (ml/kg/min)	10.7	12.6	—	—	40.4	33.1
CLhepatic (ml/kg/min)	3.1	NC	—	—	0.8	NC
B:P	2.61 (0.48)	2.50 (0.42)	—	—	1.15 (0.23)	1.12 (0.13)
AUC_liver_ (nM^*^Hr)	368,000	137,000	8,380	2,680	29,400	14,300
	0.37		0.32		0.49	
liver M:P	—	—	0.0228	0.0200	—	—
			0.88			

WT, wildtype; KO, knockout; AUC, area under the concentration-time curve, from time 0 extrapolated to infinity (blood) or until the last detectable concentration (liver); CL, clearance; M:P, metabolite:parent ratio; B:P, blood:plasma ratio; NC, not calculated. ^a^p<0.05 using student’s t-test, compared to WT. ^bb^p<0.01 using student’s t-test, compared to WT. ^ccc^p<0.001 using student’s t-test, compared to WT.

Following administration of proguanil, the exposure of cycloguanil in the blood was lower in knockout mice compared to wildtype, as shown in Figure 3A. As shown in Table 1, the metabolite:parent ratio (M:P) in the blood also decreased in knockout mice. Liver AUC of cycloguanil decreased, however the liver partitioning of cycloguanil between wildtype and knockout mice was only minorly affected following administration of proguanil (Figure 3D). The liver metabolite ratio of cycloguanil:proguanil did not change in knockout compared to wildtype mice, as shown in Figure 3E.

**FIGURE 4:**
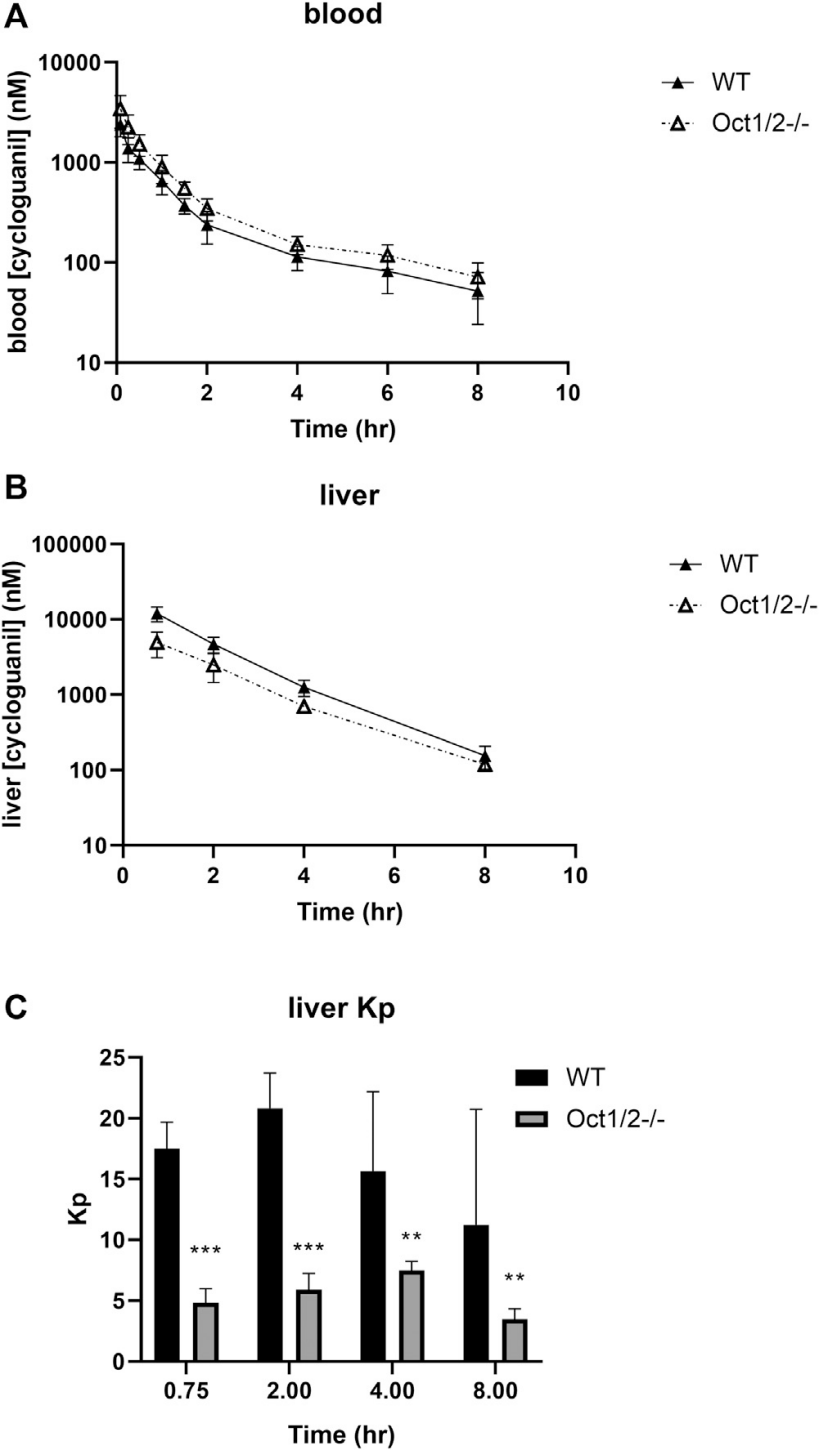
**Pharmacokinetics of cycloguanil in wildtype (WT) and Oct1/2** ^**−/−**^ **mice following IV administration of cycloguanil**. Shown are blood **(A)** and liver **(B)** concentrations for mice (*n* = 5 or 4–5/timepoint) administered cycloguanil 2 mg/kg. Filled symbols represent wildtype mice and open symbols represent knockout mice **(C)** Tissues were collected at 0.75, 2-, 4- and 8 h post-dose. Data presented as mean ± SD. **p < 0.01 using student’s t-test, compared to WT. ***p < 0.001 using student’s t-test, compared to WT. Kp = tissue:plasma partition coefficient.

Following the administration of cycloguanil, the clearance of cycloguanil changed minimally, though significantly in Oct1/2^−/−^ mice compared to wildtype (Table 1). The blood concentration-time profiles are shown in Figure 4A. Similarly to the parent proguanil, cycloguanil was found to be excreted primarily in the urine in wildtype and knockout mice and renal clearance was unchanged between the strains. Liver partitioning and exposure of cycloguanil decreased substantially, ∼3-fold in knockout compared to wildtype mice (Figures 4B,C).

**FIGURE 5:**
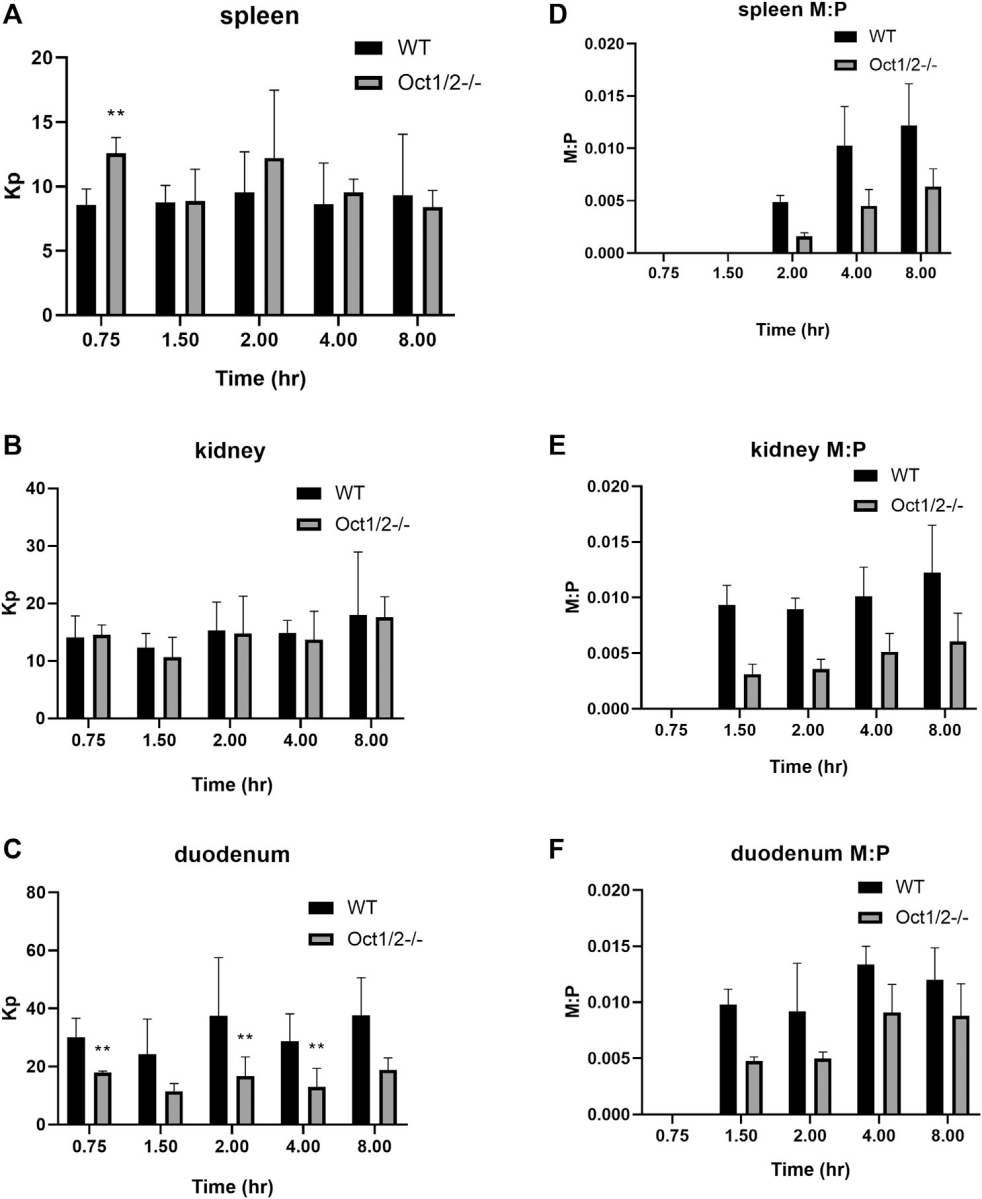
**Partitioning of proguanil (A and B, C) and cycloguanil metabolite:parent ratio (D and E, F) in tissues other than liver in wildtype (WT) and Oct1/2** ^**−/−**^ **mice following IV administration of proguanil.** Mice (*n* = 5/timepoint) were administered proguanil 2, 10 or 30 mg/kg and tissues collected at 0.75 and 2 h (2 mg/kg), 1.5 and 4 h (10 mg/kg) or 8 h (30 mg/kg) post-dose. Data presented as mean ± SD. **p < 0.01 using student’s t-test, compared to WT. Kp = tissue:plasma partition coefficient. M:P = metabolite:parent ratio.

**FIGURE 6:**
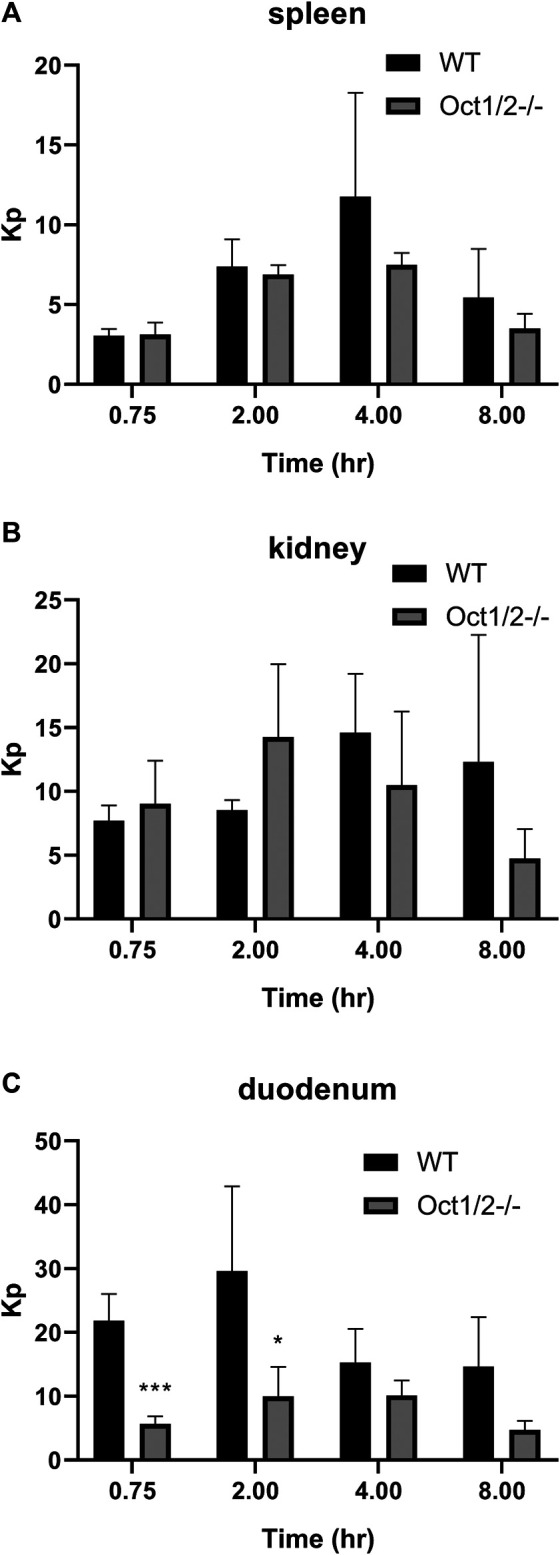
**Partitioning of cycloguanil in tissues other than liver (A and B, C) in wildtype (WT) and Oct1/2** ^**−/−**^ **mice following IV administration of cycloguanil.** Mice (*n* = 4–5/timepoint) were administered 2 mg/kg cycloguanil and sacrificed 0.75, 2, 4, or 8 h post-dose. Data presented as mean ± SD. *p < 0.05 using student’s t-test, compared to WT. ***p < 0.001 using student’s t-test, compared to WT. Kp = tissue:plasma partition coefficient.

Partitioning in organs other than the liver are shown in Figures 5,6, following proguanil and cycloguanil administration, respectively. Proguanil partitioning was decreased in the duodenum but not spleen or kidney. The cycloguanil:proguanil ratio was lower in knockout mice, at the timepoints in which cycloguanil concentrations could be detected in these tissues (Figure 5), which was consistent with the change in the systemic cycloguanil:proguanil ratio. Following cycloguanil administration, the partitioning of cycloguanil was decreased in the duodenum but not spleen or kidney, similar to proguanil.

## Discussion

There is now compelling evidence for the clinical relevance of OCT1-mediated transport in the liver, predominantly due to the extensive *in vitro* characterization of OCT1 variant activity and *in vivo* correlation with altered exposure of OCT1 substrates. In agreement with *in vitro* data generated in other laboratories, the current data with the OCT1 variants confirm the effect of the variant alleles on multiple OCT1 substrates. The substrate-dependence of OCT1*2 is particularly interesting and has been explored in detail (Seitz, 2016). Notably, the lack of effect of OCT1*2 on sumatriptan uptake has been demonstrated in the clinic, in agreement with maintained *in vitro* activity (Matthaei et al., 2015). Interestingly, however, the membrane OCT1 expression of all variants, including OCT1*2 was currently determined lower than for OCT1*1. In previous work, membrane localization of many OCT1 variants was explored qualitatively using confocal microscopy and results are again in general agreement with what we have measured using nanoLC-MS/MS (Seitz et al., 2015). Specifically, the variants which demonstrate loss-of-function across substrates, (e.g. OCT1*5 and *6) were lacking from the plasma membrane, and almost entirely localized in the endoplasmic reticulum using microscopy. We also determined that membrane expression in these variants is ∼10% of that measured for OCT1*1. Conversely, maintenance of some plasma membrane localization for variants with substrate-dependent activity, (e.g. OCT1*2 and *10) was previously reported and we determined these variants to have 25–30% of the membrane expression compare to OCT1*1 (Supplementary Table S1).

When OCT1 membrane protein was not considered, no significant difference in the sumatriptan kinetics was previously observed (Seitz, 2016), which we similarly determined. However, we observe an apparent increase in sumatriptan V_max_ when OCT1 membrane expression is considered, indicating increased intrinsic activity per mg of OCT1 protein. For fenoterol, a decrease in V_max_ without considering OCT1 protein expression was previously reported (Seitz, 2016; Tzvetkov et al., 2018), as we also determined currently. The current data indicating an apparent maintenance of activity after normalization suggests that the decrease in fenoterol activity for OCT1*2 can be explained almost entirely by membrane expression level. However, after evaluating the kinetics of fenoterol and normalizing for membrane OCT1 protein expression, it appears that the maintenance of activity is due to increased affinity, which is offset by decreased V_max_, in contrast to that of sumatriptan. Due to similarities in the kinetics of sumatriptan and fenoterol reported here and previously, prior to membrane protein normalization (Seitz, 2016), we may use previous kinetic data to speculate on the protein-normalized kinetics of other OCT1 substrates. Interestingly, while protein normalization also appeared to explain the effects of different variants on metformin uptake in the current evaluation, a substantial increase in the K_m_ and lack of change in the V_max_ for metformin in OCT1*2 has been previously reported, when membrane OCT1 protein expression is not considered (Seitz, 2016). One would assume then that the V_max_ for metformin per membrane expressed OCT1 protein must increase substantially for this variant. With regard to the effect of the variants on proguanil and cycloguanil, the current data are in agreement with the effects of OCT1*5 and *6, in that both variants have decreased uptake of both proguanil and cycloguanil. Conversely, while *2, *3 and *4 were previously reported to effect primarily uptake of proguanil, we found these variants to affect proguanil and cycloguanil similarly. For OCT1*2, we determined an apparent increase in the intrinsic activity for both proguanil and cycloguanil of ∼2-fold when considering membrane OCT1 expression. From experiments not normalized for protein expression the K_m_ and V_max_ of proguanil decreased to a similar extent (Seitz, 2016). Therefore, similar to sumatriptan, for the apparent intrinsic activity to increase when normalized for protein expression, the V_max_ for proguanil normalized to OCT1 protein would be expected to increase.

Previous data for OCT1 substrates sumatriptan and fenoterol in Oct1/2^−/−^ mice indicate changes in hepatic clearance and overall exposure consistent with that reported in humans (Morse et al., 2020). Protein expression data indicate OCT1 to be the primary OCT expressed in mice and human liver (Drozdzik et al., 2019; Morse et al., 2020). In mice, the decrease in sumatriptan and fenoterol hepatic clearance was consistently associated with a decrease in liver partitioning, which would be expected to occur in humans lacking OCT1 function as well. In humans, while proguanil was clearly demonstrated an OCT1 substrate *in vitro*, the exposure of proguanil was not altered in subjects with null OCT1 activity (Matthaei et al., 2019), which can be explained by hepatic clearance not being the major clearance pathway for proguanil. While hepatic metabolism of proguanil may be the primary route of formation of its active metabolite, it is not necessarily the primary route of elimination of the parent. Indeed, following an oral dose of proguanil, 30–69% was found in urine (Somogyi et al., 1996), meaning that urinary excretion represents at least 30–69% of proguanil elimination, depending on the bioavailability of proguanil. This appears consistent between humans and mice from the current dataset. In humans, while proguanil plasma exposure was not significantly affected, the exposure of cycloguanil was decreased in subjects with decreased OCT1 activity, with a corresponding decrease in the metabolite ratio (Matthaei et al., 2019). We observe similar effects on systemic exposure in Oct1/2^−/−^ mice.

Given that hepatocytes are a site of action/replication for malaria, an understanding of the potential liver exposures of proguanil and cycloguanil in subjects lacking OCT1 function is relevant as these may play a role in the pharmacodynamics, as discussed previously (Matthaei et al., 2019). The decrease in cycloguanil exposure indirectly supports a decrease in proguanil liver partitioning in subjects carrying OCT1 variants. The current data in mice directly indicate that proguanil liver exposure is decreased with depletion of Oct1 in mice. Given that the primary route of elimination for proguanil for humans and mice is renal clearance, it is likely that in humans the liver exposure is also decreased, as the authors of the clinical study hypothesized. The authors also determined cycloguanil to be a substrate of OCT1, therefore predicting the effect of decreased OCT1 activity on the liver exposure of cycloguanil is somewhat less straightforward. Indeed, this could mean than in subjects with null OCT1 activity, the exposure of cycloguanil may be decreased by two mechanisms, that being decreased formation due to decreased liver partitioning of proguanil, and by decreased uptake back into hepatocytes once effluxed. In the current mouse experiments, we did in fact determine lower exposure of cycloguanil in the liver of knockout mice compared to wildtype, following administration of proguanil or cycloguanil. We also confirmed that knockout of Oct1 led to decreased liver partitioning of cycloguanil, following dosing of cycloguanil. Therefore, what is unexpected in the current dataset is that when proguanil is directly administered compared to cycloguanil administration the lack of change in the metabolite:parent ratio in the liver, along with the small change in cycloguanil liver Kp in knockout compared to wildtype. This suggests that the effect of decreased OCT1 activity on liver partitioning of an OCT1 substrate that is *formed* in the liver may differ from that ascertained by assessment of that taken up into the liver. This may occur if OCT1 is responsible for both the uptake and efflux into hepatocytes, and therefore depletion of OCT1 activity may affect both to a different degree due to differences in the electrochemical gradient and concentration gradient of the substrate when a metabolite is formed vs. administered.

One potential limitation to the current dataset is the use of commercially available Oct1/2 double knockout mice, and not a model specific for Oct1. However, in our previous work, we detected only mouse Oct1 in the liver (Morse et al., 2020), suggesting that any changes in the knockout model in the liver can be attributed to changes in Oct1, not Oct2. Oct2 is highly and primarily expressed the kidney in mice, therefore given the lack of change in renal clearance, kidney partitioning and only minimal change in total clearance of either proguanil or cycloguanil, it does not appear that Oct2 knockout significantly affected the pharmacokinetics in either compound in this study. Another limitation may be measurement of membrane-associated OCT1 protein, without consideration for expression on the membrane surface. Additional techniques, such as biotinylation, may refine measurements specifically at the membrane surface to determine if membrane localization vs. surface expression may differ. Furthermore, it is difficult to confirm the results of membrane OCT1 protein in cell lines to that in hepatocytes expressing the variants, given the difficulty in identifying hepatocyte samples homozygous for all of the variants, some of which exist at very low frequency in any population (Seitz et al., 2015).

In conclusion, it is clear that there is a reproducible effect of changes in OCT1 activity *in vitro* that can be observed on plasma pharmacokinetics *in vivo*, from both human and animal studies. The mechanisms behind the altered activity and substrate-dependence have been investigated here and elsewhere. In general, from the current and previous results, even when considering differences in protein expression and affinity, the V_max_ for OCT1*2 changes in a substrate dependent manner, suggesting complex mechanisms behind activity by OCT1*2, possibilities of which have been discussed in detail (Seitz, 2016). Furthermore, the current dataset indicate that even though *in vitro* activity may reproduce changes in plasma pharmacokinetics, we are lacking in an understanding of what may be happening in sites other than plasma, particularly for metabolites that are transported by OCT1. Further work *in vitro* and *in vivo* are needed to understand these mechanisms and therefore the effects in subjects with decreased function of OCT1, especially when drug concentration at the site of action may be influenced by OCT1.

## Data Availability

The original contributions presented in the study are included in the article/Supplementary Material, further inquiries can be directed to the corresponding author.
